# Novel mutations on the *ace-1* gene of the malaria vector *Anopheles albimanus* provide evidence for balancing selection in an area of high insecticide resistance in Peru

**DOI:** 10.1186/s12936-015-0599-1

**Published:** 2015-02-14

**Authors:** Kelly A Liebman, Jesus Pinto, Jorge Valle, Miriam Palomino, Lucrecia Vizcaino, William Brogdon, Audrey Lenhart

**Affiliations:** Centers for Disease Control and Prevention, Atlanta, GA USA; Instituto Nacional de Salud, Lima, Peru; American Society for Microbiology, Washington, DC USA

**Keywords:** Insecticide resistance, Malaria, *Anopheles albimanus*, Insensitive acetylcholinesterase, *Ace-1*, Gene duplication, Balancing selection

## Abstract

**Background:**

Resistance to multiple classes of insecticides has been detected in the malaria vector *Anopheles albimanus* in northwest Peru. Acetylcholinesterase (AChE) insensitivity has previously been associated with resistance to organophosphate (OP) and carbamate (CA) insecticides in arthropods. A single point mutation on the *ace-1* gene (G119S) associated with resistance to OPs and CAs has been described previously in four anopheline species, but not in field-collected *An. albimanus.* The present study aimed to characterize the role of *ace-1* in conferring resistance to both OPs and CAs in the *An. albimanus* population in Tumbes, Peru.

**Methods:**

The frequency and intensity of resistance to OPs and CAs was quantified through bioassays of female *An. albimanus* collected between 2012 and 2014, and the presence of insensitive AChE was confirmed using biochemical assays. A portion of the *ace-1* gene flanking codon 119 was amplified and sequenced from individuals used in the bioassays and biochemical assays, as well as from historical samples collected in 2008. Statistical analyses were conducted to determine: (1) associations between genotype and AChE insensitivity; and, (2) associations between genotype and resistance phenotype.

**Results:**

After confirming high levels of resistance to fenitrothion, malathion, and bendiocarb through bioassays, two novel polymorphisms were identified at the first and second loci of codon 119, with all individuals from the 2012–2014 collections being heterozygous at the first base (G/T) and either heterozygous (G/C) or homozygous mutants (C/C) at the second base. Based on sequence data from historical samples, these mutations arose prior to 2008, but became fixed in the population between 2008 and 2012. Homozygotes at the second locus had significantly higher levels of AChE insensitivity than heterozygotes (p <0.05). Individuals phenotypically susceptible to OPs and CAs were more likely to be heterozygous at the second locus (p <0.01). Cloning identified four individuals each containing three distinct genotypes, suggesting that a duplication of the *ace-1* gene may have occurred.

**Conclusions:**

The occurrence of heterozygotes at two loci and the presence of three genotypes in four individuals suggest that balancing selection could be maintaining OP and CA resistance in this population, while minimizing associated fitness costs.

## Background

For decades, humans have used insecticides to control insect pests, including vectors of human pathogens. The widespread reliance upon and intense use of these chemicals for public health and agricultural purposes has led to the development of insecticide resistance in many insect species [1]. The biochemical and molecular pathways of resistance vary within organisms and between chemical groups, providing a complex scenario with respect to insecticide resistance management and prevention.

Organophosphates (OPs) are one of the most commonly used classes of arthropod adulticides around the world, including in Latin America. OPs and carbamates (CAs), another common insecticide, target acetylcholinesterase in insects, irreversibly inhibiting the binding of the enzyme by phosphorylation at the catalytic site [2]. An important indicator of resistance to OPs is the loss of acetylcholinesterase (AChE) sensitivity [3]. AChE is a widely distributed enzyme within the nervous system, which mediates hydrolysis of the neurotransmitter acetylcholine throughout the central and peripheral nervous systems at the postsynaptic membrane through terminating nerve impulses [4]. Inhibition of the enzyme by an OP results in the accumulation of acetylcholine, leading to signs of intoxication and finally death due to respiratory failure [5,6]. Five mutations on the *ace-1* gene were initially described in OP-resistant insects [7], but in mosquito species, only three have been linked to insensitive AChE mediated resistance: G119S, F290V and F331W [8-11]. The latter, found in *Culex tritaeniorhynchus*, changes the orientation of the catalytic histidine during hydrolysis, affecting accessibility and interaction with the H440 catalytic site [8,12]. F290V, identified originally in *Culex pipiens* from Cyprus [11], involves a substitution at the acyl binding pocket and is involved in substrate specificity [13], likely as a result of hindering the gorge entrance [14]. The most common mutation, G119S, identified in several mosquito species, occurs at the oxyanion hole of the enzyme, resulting in an amino acid change from glycine to serine leading to a steric shift, which reduces access of the insecticide to the target catalytic triad [15].

The G119S substitution has been associated with high levels of resistance in mosquito vector species, including field populations of *Anopheles gambiae* [15-18], *Anopheles coluzzii* [19] and *Culex pipiens sp.* [15,18-21]*,* as well as a laboratory strain of the malaria vector *Anopheles albimanus* [18]. The mutation appears to have arisen independently at least three times in *Cx. pipiens* [8,18,20-23], despite the presence of well-documented fitness costs to both males and females carrying the mutation. Laboratory experiments have shown that these fitness costs include longer development time, reduced overwintering survival, smaller adult size and increased risk of predation [24-28]. However, the cost may be diminished in field populations as a result of a duplication of the *ace-1* gene, which has been reported in *An. gambiae*, *An. coluzzii*, and *Cx. pipiens* [17,19,22,23,29]. Previous studies have demonstrated that having both a mutant and wild type copy of the gene can reduce fitness costs in both the presence and absence of insecticide pressure [17,22,23]. This finding is particularly troubling with respect to vector control measures, as efforts to manage resistance by removing insecticide pressure will likely not result in a full recovery of susceptibility in affected populations.

No *ace-1* mutations or duplications have been described in field populations of *An. albimanus*, the main malaria vector in many parts of Central and South America, including in Tumbes, Peru. In 2012, the Department of Health in Tumbes discontinued the use of chemical insecticides to control *An. albimanus* when it became clear that the mosquitoes were resistant to most of the major classes of insecticides used for public health purposes (Peruvian National Institute of Health, pers. comm.). However, the local *An. albimanus* population continues to be exposed to insecticides through the heavy use of agrochemicals in surrounding rice and banana plantations, and to a lesser extent through the application of insecticides to control the dengue vector, *Aedes aegypti*.

The present study aimed to further characterize the role of *ace-1* in conferring resistance to both OPs and CAs in the *An. albimanus* population in Tumbes, Peru, as *ace-1* mutations can confer cross resistance between these two chemical groups. Biochemical and bioassay data were compared with the genotype of individual mosquitoes to determine how genotypes corresponded to resistant phenotypes. The results presented herein identify two previously undescribed non-synonymous mutations at the first and second base of codon 119, which are associated with resistance and are likely maintained at high levels in the population through a duplication of the *ace-1* gene.

## Methods

### Mosquito collections

**Figure 1:**
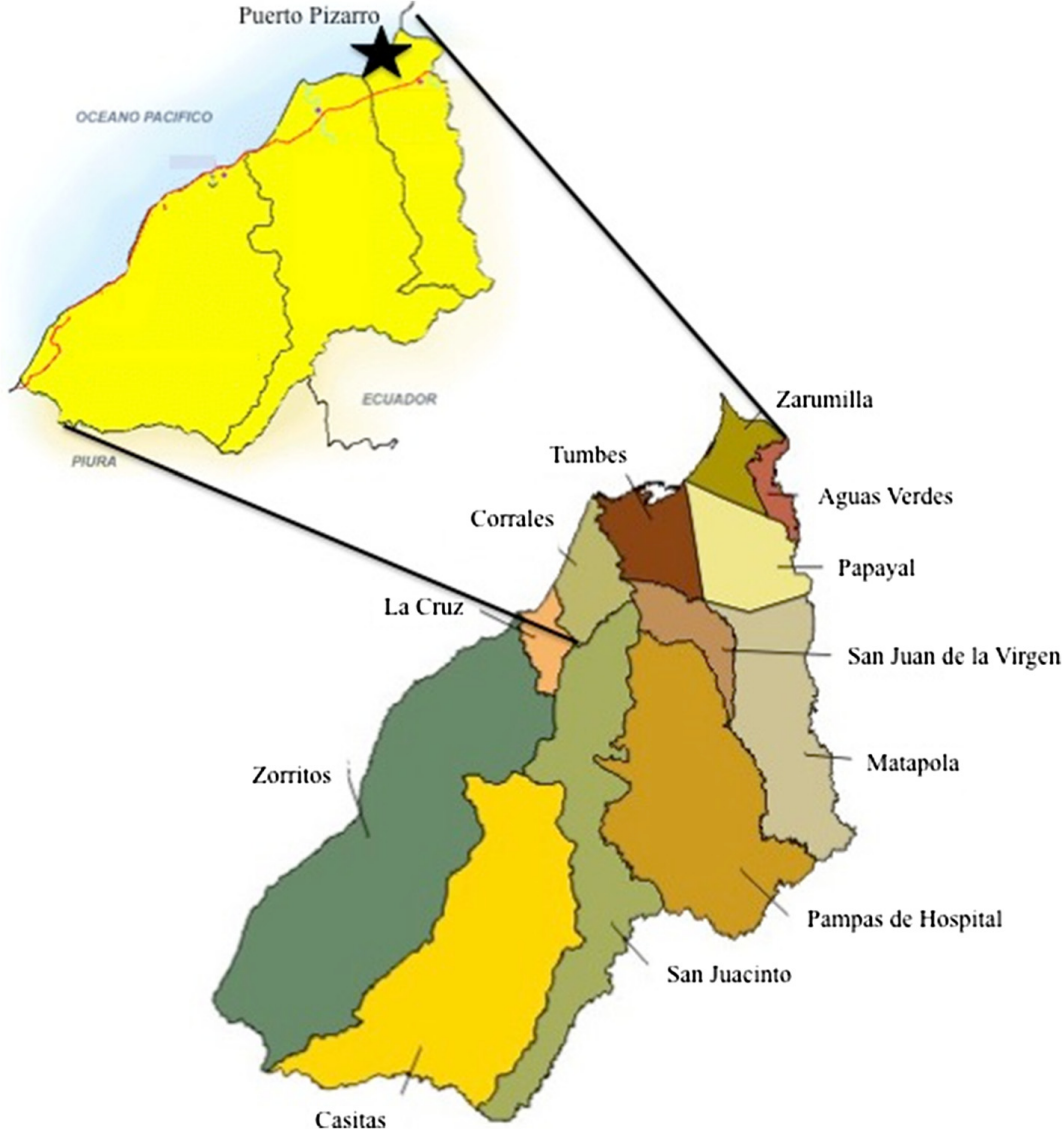
**Map indicating the study site**, **located in the Tumbes region of Peru.**

Female *An. albimanus* were collected from a family farm in Puerto Pizarro, Tumbes, Peru (3° 30' 10S, 80° 23' 38 W, Figure 1) during three sampling periods: 26–31 August, 2012 (P1), 15–19 April, 2013 (P2) and 7–10 April, 2014 (P3). Individual females were mouth aspirated from livestock corrals between 17:00 and 21:00 hours on collection days. Females collected during P1 were morphologically identified to species and immediately stored at −20°C for biochemical analyses, DNA extraction and genotyping. Females collected during P2 and P3 were morphologically identified to species, used in bioassays and subsequently separated by phenotype and stored at −20°C for DNA extraction and genotyping. Historical *An. albimanus* samples had originally been collected by the Navy Medical Research Unit – 6 (NAMRU-6) in April 2008 in Puerto Pizarro, and had either been stored in ethanol (N = 26) or dried (N = 6), and kept at −20°C until DNA extraction in 2014.

### Biochemical assays

Biochemical assays were conducted to assess AChE insensitivity [30]. Abdomens of 77 engorged *An. albimanus* females collected during P1 were excised prior to homogenization and stored for future molecular analysis. Either the full body (for mosquitoes not containing bloodmeals) or the head and thorax (for engorged mosquitoes) of the individual mosquitoes collected during P1 were homogenized in 100 uL KPO_4_ buffer (6.6 g dibasic potassium phosphate, 1.7 g monobasic potassium phosphate, 1,000 ml dH_2_O; pH = 7.2) and then diluted to 100 uL with additional KPO_4_ buffer. As a negative control, the same homogenization procedure was followed with mosquitoes from the Sanarate insecticide-susceptible laboratory strain of *An. albimanus*. To determine the insensitive AChE activity levels, 100 ul of this homogenate was added in triplicate to wells on a 96-well plate, followed by 100 ul ATCH (75 mg acetyl-thio-choline iodide, 21 mg propoxur, 10 ml acetone, 90 ml KPO_4_ buffer), then 100 ul DTNB (13 mg Dithio-bis-2-nitrobenzoic acid, 100 ml KPO_4_ buffer). The plate was read immediately using 414-nm filter on a SpectraMax M5^e^ plate reader (Molecular Devices), then again at 10 min using the same filter. The difference between the two readings was the value used for statistical analysis.

### Bottle bioassays

CDC bottle bioassays [31] were conducted on female *An. albimanus* collected during P2 and P3. A modification of the CDC bottle bioassay in which mosquitoes were exposed to one, two, five and ten times the diagnostic dose of insecticide was used to assess resistance intensity. Results were interpreted using the updated WHO criteria: 98-100% mortality at 30 min indicates susceptibility, 90-97% mortality suggests the possibility of resistance and <90% mortality suggests the presence of resistance [32].

During P2, bottles were treated with 50 (diagnostic dose), 100, 250 or 500 ug/bottle of either fenitrothion or malathion, two commonly used OPs. During P3, fenitrothion was tested as during P2, and a standard bottle bioassay for the CA bendiocarb was conducted, using the diagnostic dose of 50 ug/bottle. Each replicate also included an untreated control bottle. For both periods, bioassays were run with two different endpoints: (1) mosquitoes were exposed to the insecticide for 120 min with recordings of knock down at 15-min intervals; and, (2) mosquitoes were exposed for 30 min and then removed, classified as susceptible or resistant, and used for molecular analyses.

### DNA extraction and *ace-1* amplification

DNA was extracted from the excised abdomens of the mosquitoes collected during P1 using the method described by Collins *et al.* [33], and samples were eluted in 25 uL of TE buffer. The full bodies of the historical samples as well as the mosquitoes collected during P2 and P3 were homogenized in 180 uL phosphate buffered saline (PBS) and extracted using DNeasy Blood and Tissue Kits (Qiagen) per the manufacturer’s instructions, and were eluted to a final volume of 100 uL. All extracted samples were stored at −20°C prior to DNA amplification.

*Anopheles albimanus-*specific primers were designed to amplify the region of the *ace-1* gene containing codon 119 (AAace1F 5’ TGTGGAACCCAAATACGC’3 and AAace1R 5‘ACGTTCTCTTCCGAGGCG’3). The PCR reaction contained a final volume of 25 uL: 2.5 uL 5X *Taq* DNA polymerase buffer (Promega), 250 uM deoxynucleoside triphosphate, 1U *Taq* DNA polymerase (Promega), 10 pmol of each primer and 50 ng of DNA template. Cycling conditions included an initial denaturation step at 94°C for 5 min, followed by 30 cycles of 94°C for 30 sec, 60°C for 30 sec and 72°C for 60 sec, and a final extension at 72°C for 5 min. Amplicons were visualized by electrophoresis on a 2% agarose gel stained with ethidium bromide.

### Sequencing and cloning

Amplicons were purified using a Multiscreen PCR 96-well plate (Millipore) per the manufacturer’s instructions and were sequenced directly using primers AAace1F and AAace1R on an Applied Biosystems 3500xL Genetic Analyzer (Applied Biosystems). Purification prior to sequencing was performed using Applied Biosystems BigDye XTerminator Purification Kit, per manufacturer’s instructions (Applied Biosystems). Genotypes were analysed using SeqMan Pro, a component of the DNASTAR LaserGene 11.0 suite. Three phenotypically susceptible individuals (KCC, KSC(1) and KSC(2)) and three phenotypically resistant individuals (KSC(3), KSC(4), KSC(5)) were selected for cloning, using the pGem cloning kit (Promega) and following the manufacturer’s protocol. Between seven and 15 clones for each individual were amplified, purified, sequenced directly, and analysed.

### Data analyses

#### Genotype and biochemical assay

T-tests were conducted to determine if an association existed between insensitive AChE activity and the genotype of individual mosquitoes collected during P1.

### Genotype and phenotype

Independent Chi-square tests were conducted to identify associations between susceptible and resistant phenotypes and the genetic profile at codon 119 of individual mosquitoes collected during P2 and P3. All statistical analyses were conducted using the R project for statistical computing.

## Results

### Biochemical analyses

**Figure 2:**
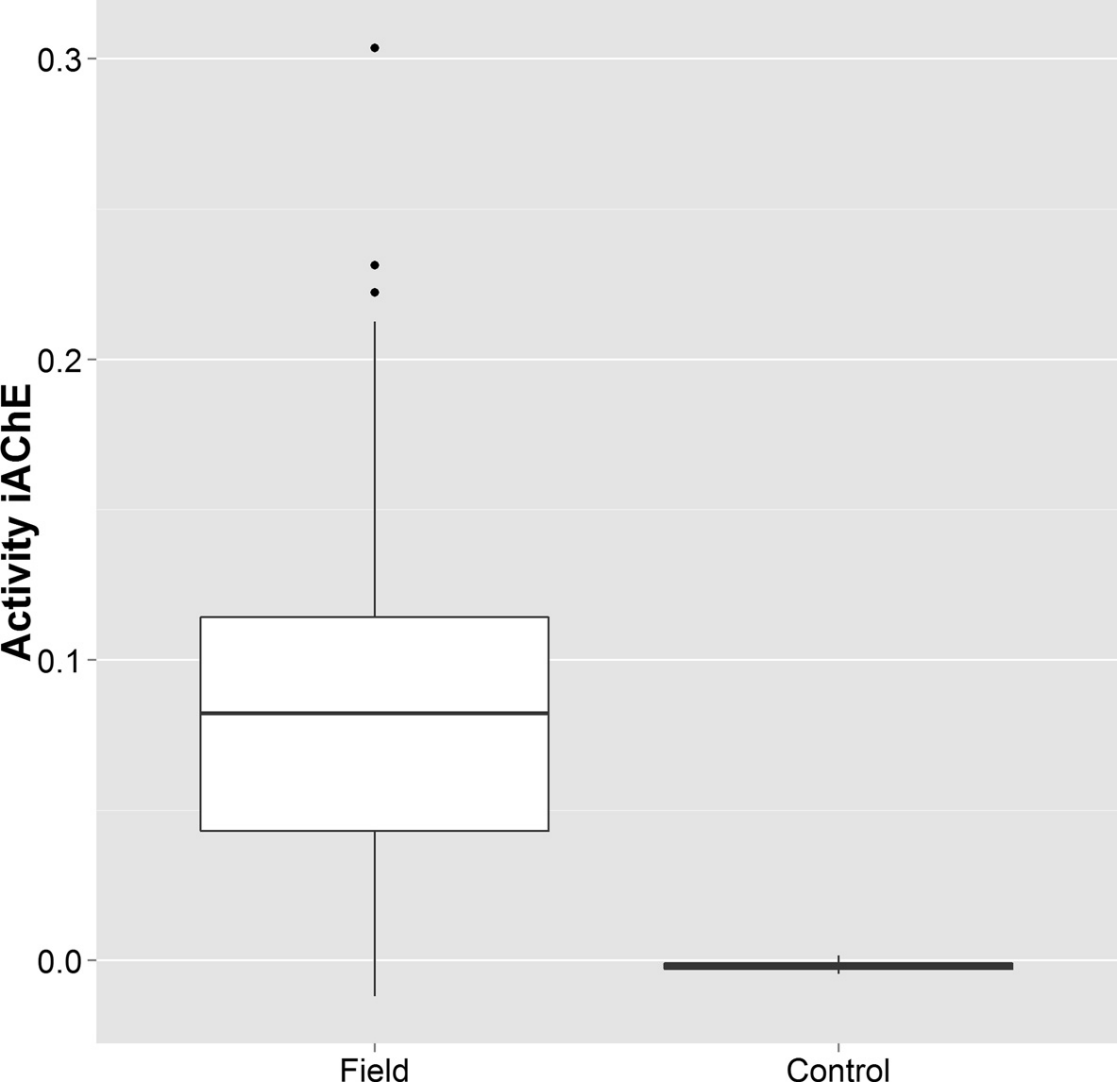
**Analysis of acetylcholinesterase insensitivity in** ***Anopheles albimanus*** **collected in 2012 from Tumbes,** **Peru as compared to the Sanarate** **-susceptible** ***Anopheles albimanus*** **strain** **(control),** **as measured by AChE activity in the presence of an inhibitor (propoxur).**

Significant AChE insensitivity was detected in the field-collected mosquitoes (mean change in absorbance = 0.083), as compared with the Sanarate-susceptible control strain (mean = 0.00, p <0.01, Figure 2).

### Intensity bottle bioassays

**Figure 3:**
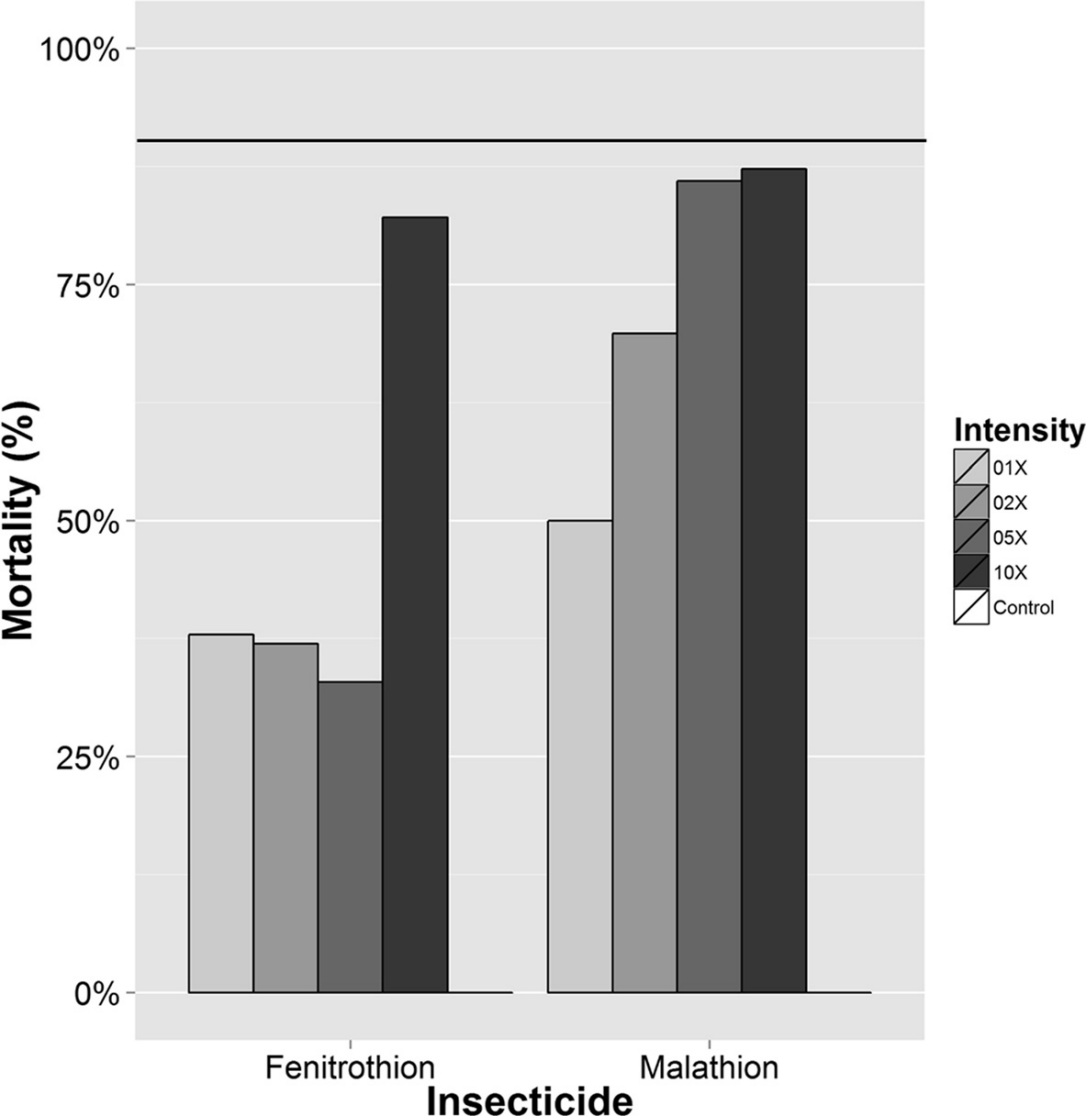
**Results of bottle bioassays for malathion and fenitrothion** **(P2,** **2013).** Percent mortality at the diagnostic time (30 min) to the diagnostic dose and multiples thereof.

During P2, 272 mosquitoes were exposed to varying doses of fenitrothion (1× the diagnostic dose, n = 58; 2× the diagnostic dose, n = 65; 5× the diagnostic dose, n = 82 and 10× the diagnostic dose, n = 67), and 229 were exposed to varying doses of malathion (1×, n = 64; 2×, n = 53; 5×, n = 57 and 10×, n = 55). Bioassay results showed high levels of both resistance frequency and intensity for both OPs in this *An. albimanus* population (Figure 3).

**Figure 4:**
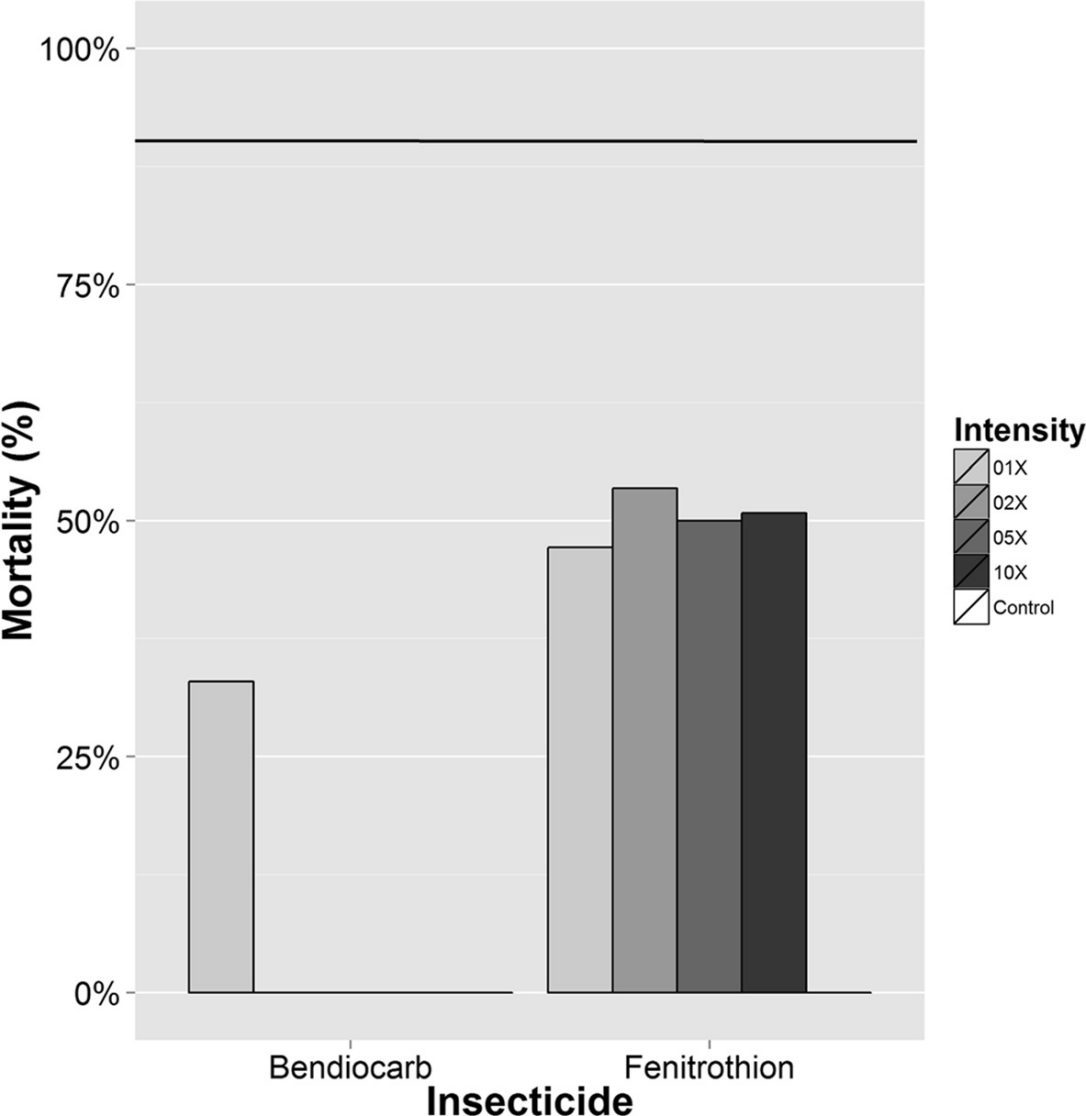
**Results of bottle bioassays for fenitrothion and bendiocarb** **(P3,** **2014).** Percent mortality at the diagnostic time (30 min) to the diagnostic dose of bendiocarb and multiples of the diagnostic dose of fenitrothion.

During P3, 282 mosquitoes were exposed to varying doses of fenitrothion (1×, n = 70; 2×, n = 73; 5×, n = 76; 10×, n = 63). As during P2, resistance was confirmed at ten times the diagnostic dose of fenitrothion, but had increased from 82.1% mortality in P2 to 50.8% in P3 (Figure 4). Interestingly, resistance across all intensity levels was even (approximately 50% mortality), suggesting that the resistance in this population, although not uniformly present, is of great intensity. Ninety-seven mosquitoes were exposed to the diagnostic dose of bendiocarb, identifying significant resistance to this insecticide (33% mortality, Figure 4).

### *Ace-1* amplification, sequencing, and cloning

The region including codon 119 of the *ace-1* gene was amplified from 487 female *An. albimanus*: 32 from 2008, 77 from P1, 188 from P2 (79 susceptible to fenitrothion, 39 resistant to fenitrothion, 55 susceptible to malathion and 15 resistant to malathion) and 190 from P3 (38 susceptible to fenitrothion, 52 resistant to fenitrothion, 35 susceptible to bendiocarb and 65 resistant to bendiocarb). The resulting 139 bp amplicons were purified and sequenced to determine genetic profile.

Sequencing of historical samples from 2008 yielded four distinct profiles: wild type (WT; GGC, coding for glycine), second base heterozygotes (GSC, coding for either WT glycine or alanine; S = G/C), first and second base heterozygotes (KSC, coding for glycine, serine, alanine, or cysteine; K = G/T) and first base heterozygote/second base mutant (KCC, coding for serine or alanine) (Table 1). This is the first report of a non-synonymous mutation at either the first or second base of codon 119 in *An. albimanus*. Sequencing of individuals from P1, P2 and P3 identified only two of these genotypes: KSC and KCC, and all individuals were heterozygous at the first base (Table 1). The proportion of mosquitoes homozygous mutant at the second base increased significantly from 2012 to 2013 (from 18.2 to 33%, p = 0.02).

**Table 1:** **Total number and percentage of each genotype at codon 119 from the population sampled during each year**; **K** = **G**/**T**, **S** = **G**/**C**

**Genotype**	**2008**	**2012** ** (P1)**	**2013** **(P2)**	**2014** ** (P3)**
GGC (WT)	4 (12.5%)	0 (0%)	0 (0%)	0 (0%)
KSC	22 (68.8%)	63 (81.8%)	126 (67.0%)	136 (71.6%)
KCC	4 (12.5%)	14 (18.2%)	62 (33.0%)	54 (28.4%)
GSC	2 (6.3%)	0 (0%)	0 (0%)	0 (0%)
Total	32	77	188	190

Clones were sequenced from one individual that was homozygous mutant at the second base and five individuals that were heterozygous at the second base, identifying three genotypes at codon 119 (Table 2). For the second-base homozygous individual, six clones coded for serine at position 119, the known resistant genotype, and two coded for alanine. The first susceptible second-base heterozygous individual (KSC(1)) yielded four (WT) glycine clones and three serine clones. The second phenotypically susceptible second-base heterozygote (KSC(2)) and all of the phenotypically resistant individuals (KSC(3), KSC(4), KSC(5)) yielded clones of glycine, serine and alanine (Table 2). The detection of three distinct genotypes in four individuals suggests that a duplication of the *ace-1* gene has occurred in this population of *An. albimanus*.

**Table 2:** **Codon 119 genotypes of three cloned phenotypically susceptible** (**KCC**, **KSC**(**1**), **KSC**(**2**)) **and three phenotypically resistant** (**KSC**(**3**), **KSC**(**4**), **KSC**(**5**)) ***Anopheles albimanus***

	**Clone genotypes**			
**Original sequence**	**GGC** **(Gly, ** **WT)**	**TCC (Ser, Resistant)**	**GCC (Ala)**	**TGC (Cys)**
KCC	0	6	2	0
KSC(1)	4	3	0	0
KSC(2)	5	4	2	0
KSC(3)	3	9	3	0
KSC(4)	3	8	3	0
KSC(5)	4	6	3	0

### Data analyses

#### Genotype and AChE insensitivity

**Figure 5:**
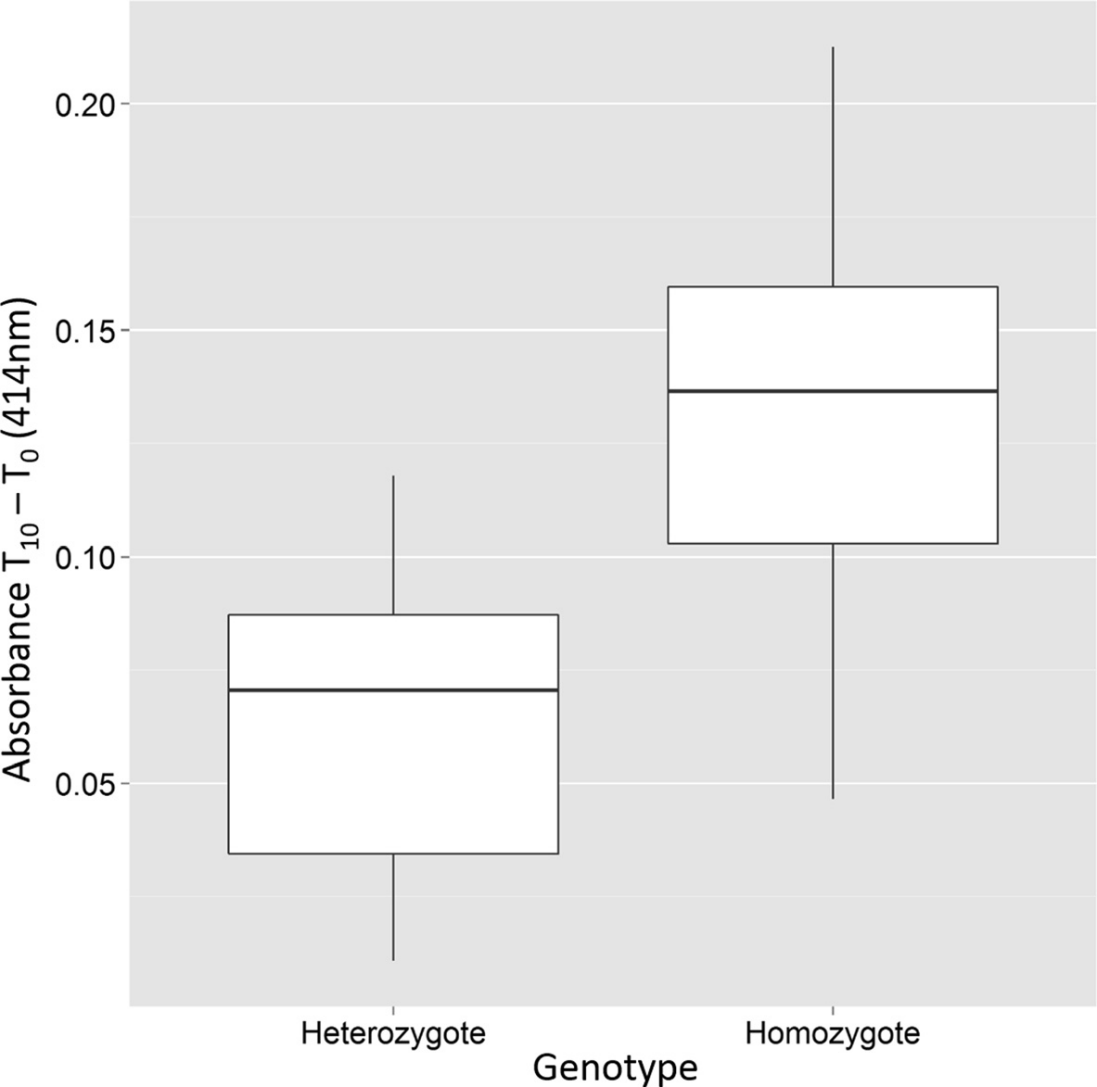
**Average activity of insensitive acetylcholinesterase** **(iAChE)** **for individuals that were heterozygous** **(G/** **C)** ***vs*** **homozygous** **(C/** **C)** **at the second base of codon 119.** Significantly greater insensitivity was detected in homozygotes (p < 0.05).

T-tests comparing the activity of insensitive AChE in KSC (second base heterozygote) and KCC (second base homozygote mutant) individuals showed significantly higher AChE insensitivity in KCC individuals (Figure 5, p <0.05).

### Phenotype and genotype

Chi-square tests conducted on genotype and bioassay data from P2 samples indicated that significantly more individuals susceptible to any dose of malathion or fenitrothion were heterozygous at the second base, suggesting that heterozygotes at both bases may have increased susceptibility (Table 3a, p <0.05). The relatively equal proportion of resistant individuals that were heterozygous or homozygous at the second locus indicates that the novel polymorphism may confer increased susceptibility, though it does not appear to increase resistance.

**Table 3:** **Genotype at the second locus of codon 119 by resistance phenotype, for (a) P2 (malathion and fenitrothion), (b) P3 (fenitrothion) (c) by intensity of resistance to fenitrothion in P3 and (d) bendiocarb, P3**

**Phenotype**	**Heterozygote (G/C)**	**Homozygote (C/C)**	**Total**
**(a)**			
Susceptible	99(73.9%)	35(26.1%)	134(100%)
Resistant	27(50.0%)	27(50.0%)	54(100%)
**(b)**			
Susceptible	34(89.5%)	4(10.5%)	38(100%)
Resistant	31(59.6%)	21(40.4%)	52(100%)
**(c)**			
1x diagnostic dose			
Susceptible	3(75.0%)	1(25.0%)	4(100%)
Resistant	12(63.2%)	7(36.8%)	19(100%)
2x diagnostic dose			
Susceptible	6(100%)	0(0%)	6(100%)
Resistant	10(62.5%)	6(37.5%)	16(100%)
5x diagnostic dose			
Susceptible	11(84.6%)	2(15.4%)	13(100%)
Resistant	6(60.0%)	4(40.0%)	10(100%)
10x diagnostic dose			
Susceptible	14(93.3%)	1(6.7%)	15(100%)
Resistant	3(42.9%)	4(57.1%)	7(100%)
**(d)**			
Susceptible	30(85.7%)	5(14.3%)	35(100%)
Resistant	41(63.1%)	24(36.9%)	65(100%)

Identical analyses were conducted with the P3 samples, and similar results were obtained for all intensities of fenitrothion (Table 3b and c, p < 0.05) and the diagnostic dose of bendiocarb (Table 3d, p <0.05). When the same individuals from the fenitrothion assays were analysed with respect to the intensity of resistance, the same pattern held true, with the exception of the diagnostic dose, in which susceptible individuals were statistically equally likely to be heterozygous or homozygous at the second locus (Table 3c, p = 0.317). However, this result could be due to the low sample size (n = 4) for susceptible individuals at this dose.

## Discussion

OP and CA insecticides have been used for many years around the world for the control of malaria vectors. With OP and CA resistance now being reported in several important vector species, understanding the mechanisms underlying this resistance is essential to guide the use of these chemicals and preserve their efficacy as vector control tools. Depending on how resistance arises, discontinuing the use of OPs and CAs may result in a return to susceptibility in the population over time [24,25,34,35]. The results of this study suggest that *An. albimanus* is adapting to maintain high levels of resistance to OPs and CAs in Tumbes, Peru, providing a worrisome scenario from a resistance management perspective.

Resistance to OPs and CAs in *An. albimanus* has previously been associated with insensitive AChE [30,36-39]. The G119S mutation had previously been reported in an OP-resistant laboratory strain of *An. albimanus* [15], but in the Tumbes population, this serine substitution was not related to the previously reported single point mutation. Instead, the high degree of OP and CA resistance was associated with two previously undescribed polymorphisms at the first and second loci of codon 119. The polymorphism at the first locus, G/G to G/T, arose prior to 2008 and has subsequently become fixed in the population. The G-C substitution at the second locus also appeared prior to 2008, and may possibly be moving toward fixation, with an increase in C homozygotes from 18.2 to 28.2% detected between 2012 and 2014. Data from historical samples suggest that this novel substitution at the second locus may have arisen prior to the substitution at the first locus, which is in contrast to the findings of previous studies, which have only identified a mutation at the first locus.

The majority of individuals from the 2008 collection (~80%) were heterozygous at the first base of codon 119. However, the genotypes of two individuals from 2008 were heterozygous at base 2, but homozygous wild type at base 1. This was unexpected, as the possible amino acids for these individuals are glycine (susceptible) and alanine, rather than the amino acid associated with resistance, serine. Serine confers resistance by altering the conformation of the protein and reducing access to the catalytic triad [18]. If alanine had the same effect, it can be assumed that all individuals that were heterozygous at base 1 and homozygous mutant at base 2 would be resistant to OPs and, likely, CAs. However, in both 2013 and 2014, there were individuals homozygous mutant at base 2 and heterozygous at base 1 that were phenotypically susceptible to fenitrothion, malathion and bendiocarb, indicating that any structural changes associated with alanine may not confer the same level of resistance as the serine mutation.

Since 2012, the mutant allele at the first base of codon 119 has become fixed in the population, with 100% of samples heterozygous at this locus. The maintenance of this polymorphism in the population is likely due to a duplication of the *ace-1* gene, which has previously been reported in the malaria vectors *An. gambaie, An. coluzzii* and *Cx. pipiens* [17,22,29,40]. In the present study, the detection of three genotypes in four individuals provides the first evidence that an *ace-1* gene duplication may have occurred in a field population of *An. albimanus.* If present, this duplication would allow individuals to have both susceptible and resistant copies of the gene, which likely decreases fitness costs associated with the resistant genotype. This has been observed in *Cx. pipiens* populations that have undergone a recent *ace-1* mutation and duplication event [22,23,41], and has likely evolved in nature through balancing selection. This evolutionary mechanism, which has recently been implicated in the maintenance of other alleles associated with insecticide resistance [42], allows for the maintenance of genetic polymorphisms in a population while minimizing associated fitness costs. In such a scenario, heterozygotes have an advantage in that they contain both the resistant and wild type alleles, and as such, they are able to survive insecticide exposure but at a reduced cost to their overall fitness when compared with individuals that have only the resistant allele.

The intensive use of agrochemicals in the Tumbes region will likely provide this population with constant exposure to a variety of insecticides. The role that these agricultural chemicals play in the development and maintenance of resistance has been discussed with respect to African malaria vectors [43-45], and similar scenarios are clearly present in Latin America as well. The rotation of different chemicals throughout the year to control various crop pests results in the inadvertent exposure of malaria vectors to different insecticide groups, often at low and/or sub-lethal doses. Unfortunately, the regulation of agricultural insecticide use is challenging to implement and enforce, and it therefore seems unlikely that this source of insecticide selection pressure will change.

## Conclusions

This study presents the first evidence of two previously undescribed mutations associated with high levels of resistance to OP and CA insecticides in a field population of *An. albimanus,* potentially maintained in the population by a duplication of the *ace-1* gene. Data from historical samples suggest that the mutation at the second locus of codon 119 may have arisen prior to the mutation at the first locus. From 2012 to 2014 there was an increase in the number of homozygotes at the second locus, indicating that the mutant allele may be moving toward fixation in the population. If this is the case, the wild type susceptible genotype will no longer exist in this population and resistance will likely be irreversible. In the absence of efforts to decrease the insecticide pressure on this population, its susceptibility to two key classes of chemical insecticides used for malaria vector control is likely to be lost.

## References

[1] Bills P (2001). A new database of pesticide resistant insects and mites (Arthropods). Pesticide notes.

[2] Aldridge WN (1950). Some properties of specific cholinesterase with particular reference to the mechanism of inhibition by diethyl p-nitrophenyl thiophosphate (E 605) and analogues. Biochem J.

[3] Ayad H, Georghiou P (1975). Resistance to organophosphates and carbamates in *Anopheles albimanus* based on reduced sensitivity of acetylcholinesterase. J Econ Entomol.

[4] Grisaru D, Sternfeld M, Eldor A, Glick D, Soreq H (1999). Structural roles of acetylcholinesterase variants in biology and pathology. Eur J Biochem.

[5] Chambers JE, Levi PE (1992). Organophosphates: Chemistry, Fate and Effects.

[6] Chambers JE, Meek EC, Chambers HW, Krieger R (2010). The metabolism of organophosphorus insecticides. Hayes' Handbook of Pesticide Toxicology.

[7] Mutero A, Pralavorio M, Bride JM, Fournier D (1994). Resistance-associated point mutations in insecticide-insensitive acetylcholinesterase. Proc Natl Acad Sci U S A.

[8] Alout H, Berthomieu A, Cui F, Tan Y, Berticat C, Qiao C (2007). Different amino-acid substitutions confer insecticide resistance through acetylcholinesterase 1 insensitivity in *Culex vishnui* and *Culex tritaeniorhynchus* (Diptera: Culicidae) from China. J Med Entomol.

[9] Djogbenou L, Dabire R, Diabate A, Kengne P, Akogbeto M, Hougard JM (2008). Identification and geographic distribution of the ACE-1R mutation in the malaria vector anopheles gambiae in south-western Burkina Faso, west africa. Am J Trop Med Hyg.

[10] Nabeshima T, Mori A, Kozaki T, Iwata Y, Hidoh O, Harada S (2004). An amino acid substitution attributable to insecticide-insensitivity of acetylcholinesterase in a Japanese encephalitis vector mosquito, *Culex tritaeniorhynchus*. Biochem Biophys Res Commun.

[11] Alout H, Berthomieu A, Hadjivassilis A, Weill M (2007). A new amino-acid substitution in acetylcholinesterase 1 confers insecticide resistance to *Culex pipiens* mosquitoes from Cyprus. Insect Biochem Mol Biol.

[12] Millard CB, Koellner G, Ordentlich A, Shafferman A, Silman I, Sussman JL (1999). Reaction products of acetylcholinesterase and VX reveal a mobile histidine in the catalytic triad. J Am Chem Soc.

[13] Alout H, Weill M (2008). Amino-acid substitutions in acetylcholinesterase 1 involved in insecticide resistance in mosquitoes. Chem Biol Interact.

[14] Harel M, Sussman JL, Krejci E, Bon S, Chanal P, Massoulie J (1992). Conversion of acetylcholinesterase to butyrylcholinesterase: modeling and mutagenesis. Proc Natl Acad Sci U S A.

[15] Weill M, Malcolm C, Chandre F, Mogensen K, Berthomieu A, Marquine M (2004). The unique mutation in ace-1 giving high insecticide resistance is easily detectable in mosquito vectors. Insect Mol Biol.

[16] Djogbenou L, Chandre F, Berthomieu A, Dabire R, Koffi A, Alout H (2008). Evidence of introgression of the ace-1(R) mutation and of the ace-1 duplication in west African anopheles gambiae ss. PLoS One.

[17] Edi CV, Djogbenou L, Jenkins AM, Regna K, Muskavitch MA, Poupardin R (2014). CYP6 P450 enzymes and ACE-1 duplication produce extreme and multiple insecticide resistance in the malaria mosquito *Anopheles gambiae*. PLoS Genet.

[18] Weill M, Lutfalla G, Mogensen K, Chandre F, Berthomieu A, Berticat C (2003). Comparative genomics: Insecticide resistance in mosquito vectors. Nature.

[19] Essandoh J, Yawson AE, Weetman D (2013). Acetylcholinesterase (Ace-1) target site mutation 119S is strongly diagnostic of carbamate and organophosphate resistance in anopheles gambiae ss and anopheles coluzzii across southern Ghana. Malar J.

[20] Cui F, Raymond M, Berthomieu A, Alout H, Weill M, Qiao CL (2006). Recent emergence of insensitive acetylcholinesterase in Chinese populations of the mosquito *Culex pipiens* (diptera: culicidae). J Med Entomol.

[21] Djogbenou L, Akogbeto M, Chandre F (2008). Presence of insensitive acetylcholinesterase in wild populations of *Culex pipiens quinquefasciatus* from Benin. Acta Trop.

[22] Labbe P, Berthomieu A, Berticat C, Alout H, Raymond M, Lenormand T (2007). Independent duplications of the acetylcholinesterase gene conferring insecticide resistance in the mosquito *Culex pipiens*. Mol Biol Evol.

[23] Labbe P, Berticat C, Berthomieu A, Unal S, Bernard C, Weill M (2007). Forty years of erratic insecticide resistance evolution in the mosquito *Culex pipiens*. PLoS Genet.

[24] Berticat C, Boquien G, Raymond M, Chevillon C (2002). Insecticide resistance genes induce a mating competition cost in *Culex pipiens* mosquitoes. Genet Res.

[25] Berticat C, Duron O, Heyse D, Raymond M (2004). Insecticide resistance genes confer a predation cost on mosquitoes, *Culex pipiens*. Genet Res.

[26] Bourguet D, Guillemaud T, Chevillon C, Raymond M (2004). Fitness costs of insecticide resistance in natural breeding sites of the mosquito *Culex pipiens*. Evolution.

[27] Djogbenou L, Noel V, Agnew P (2010). Costs of insensitive acetylcholinesterase insecticide resistance for the malaria vector *Anopheles gambiae* homozygous for the G119S mutation. Malar J.

[28] Gazave E, Chevillon C, Lenormand T, Marquine M, Raymond M (2001). Dissecting the cost of insecticide resistance genes during the overwintering period of the mosquito *Culex pipiens*. Heredity (Edinb).

[29] Djogbenou L, Labbe P, Chandre F, Pasteur N, Weill M (2009). Ace-1 duplication in *Anopheles gambiae*: a challenge for malaria control. Malar J.

[30] Brogdon WG, Beach RF, Stewart JM, Castanaza L (1988). Microplate assay analysis of the distribution of organophosphate and carbamate resistance in Guatemalan *Anopheles albimanus*. Bull World Health Organ.

[31] Brogdon WG, Chan A (2010). Guideline for evaluating insecticide resistance in vectors using the CDC bottle bioassay.

[32] World Health Organization W (2013). Test procedures for insecticide resistance monitoring in malaria vector mosquitoes.

[33] Collins FH, Maendez MAR MO, Mehaffey PC, Besansky NJ, Finnerty V (1987). A ribosomal RNA gene probe differentiates member species of the *Anopheles gambiae* complex. Am J Trop Med Hyg.

[34] Alout H, Djogbenou L, Berticat C, Chandre F, Weill M (2008). Comparison of *Anopheles gambiae* and *Culex pipiens* acetycholinesterase 1 biochemical properties. Comp Biochem Physiol B Biochem Mol Biol.

[35] Mouches C, Pasteur N, Berge JB, Hyrien O, Raymond M, de Saint Vincent BR (1986). Amplification of an esterase gene is responsible for insecticide resistance in a California Culex mosquito. Science.

[36] Dary O, Georghiou GP, Parsons E, Pasteur N (1991). Dot-blot test for identification of insecticide-resistant acetylcholinesterase in single insects. J Econ Entomol.

[37] Ffrench-Constant RH, Bonning BC (1989). Rapid microtitre plate test distinguishes insecticide resistant acetylcholinesterase genotypes in the mosquitoes *Anopheles albimanus, An. nigerrimus* and *Culex pipiens*. Med Vet Entomol.

[38] Hemingway J, Georghiou GP (1983). Studies on the acetylcholinesterase of *Anopheles albimanus* resistant and susceptible to organophosphate and carbamate insecticides. Pestic Biochem Physiol.

[39] Penilla RP, Rodriguez AD, Hemingway J, Torres JL, Arredondo-Jimenez JI, Rodriguez MH (1998). Resistance management strategies in malaria vector mosquito control. Baseline data for a large-scale field trial against *Anopheles albimanus* in Mexico. Med Vet Entomol.

[40] Fournier D (2005). Mutations of acetylcholinesterase which confer insecticide resistance in insect populations. Chem Biol Interact.

[41] Lenormand T, Bourguet D, Guillemaud T, Raymond M (1999). Tracking the evolution of insecticide resistance in the mosquito *Culex pipiens*. Nature.

[42] Martins AJ, Brito LP, Linss JG, Rivas GB, Machado R, Bruno RV (2013). Evidence for gene duplication in the voltage-gated sodium channel gene of *Aedes aegypti*. Evol Med Public Health.

[43] Chouaibou M, Etang J, Brevault T, Nwane P, Hinzoumbe CK, Mimpfoundi R (2008). Dynamics of insecticide resistance in the malaria vector *Anopheles gambiae s.l*. from an area of extensive cotton cultivation in Northern Cameroon. Trop Med Int Health.

[44] Dabire KR, Diabate A, Djogbenou L, Ouari A, N'Guessan R, Ouedraogo JB (2008). Dynamics of multiple insecticide resistance in the malaria vector *Anopheles gambiae* in a rice growing area in South-Western Burkina Faso. Malar J.

[45] Diabate A, Baldet T, Chandre F, Akoobeto M, Guiguemde TR, Darriet F (2002). The role of agricultural use of insecticides in resistance to pyrethroids in *Anopheles gambiae s.l.* in Burkina Faso. Am J Trop Med Hyg.

